# Effects of photon irradiation in the presence and absence of hindlimb unloading on the behavioral performance and metabolic pathways in the plasma of Fischer rats

**DOI:** 10.3389/fphys.2023.1316186

**Published:** 2024-01-08

**Authors:** Jacob Raber, Sarah Holden, Kat Kessler, Breanna Glaeser, Chloe McQuesten, Mitali Chaudhari, Fiona Stenzel, Marek Lenarczyk, Scott Willem Leonard, Jeffrey Morré, Jaewoo Choi, Amy Kronenberg, Alexander Borg, Andy Kwok, Jan Frederik Stevens, Christopher Olsen, Jeffrey S. Willey, Gerd Bobe, Jessica Minnier, John E. Baker

**Affiliations:** ^1^ Department of Behavioral Neuroscience, Oregon Health & Science University, Portland, OR, United States; ^2^ Departments of Neurology, and Radiation Medicine, Division of Neuroscience ONPRC, Oregon Health & Science University, Portland, OR, United States; ^3^ College of Pharmacy, Oregon State University, Corvallis, OR, United States; ^4^ Neuroscience Center and Department of Pharmacology and Toxicology, Medical College of Wisconsin, Milwaukee, WI, United States; ^5^ Radiation Biosciences Laboratory, Medical College of Wisconsin, Milwaukee, WI, United States; ^6^ Department of Radiation Oncology, Wake Forest School of Medicine, Winston-Salem, NC, United States; ^7^ Mass Spectrometry Core, Oregon State University, Corvallis, OR, United States; ^8^ Linus Pauling Institute, Oregon State University, Corvallis, OR, United States; ^9^ Biological Systems and Engineering Division, Lawrence Berkeley National Laboratory, Berkeley, CA, United States; ^10^ Department of Animal Sciences, Oregon State University, Corvallis, OR, United States; ^11^ Oregon Health & Science University-Portland State University School of Public Health, Knight Cancer Institute Biostatistics Shared Resource, The Knight Cardiovascular Institute, OR Health & Science University, Portland, OR, United States

**Keywords:** Fischer, rats, photon irradiation, hindlimb unloading, open field, anhedonia, elevated plus maze, metabolomics

## Abstract

**Introduction:** The space environment astronauts experience during space missions consists of multiple environmental challenges, including microgravity. In this study, we assessed the behavioral and cognitive performances of male Fisher rats 2 months after sham irradiation or total body irradiation with photons in the absence or presence of simulated microgravity. We analyzed the plasma collected 9 months after sham irradiation or total body irradiation for distinct alterations in metabolic pathways and to determine whether changes to metabolic measures were associated with specific behavioral and cognitive measures.

**Methods:** A total of 344 male Fischer rats were irradiated with photons (6 MeV; 3, 8, or 10 Gy) in the absence or presence of simulated weightlessness achieved using hindlimb unloading (HU). To identify potential plasma biomarkers of photon radiation exposure or the HU condition for behavioral or cognitive performance, we performed regression analyses.

**Results:** The behavioral effects of HU on activity levels in an open field, measures of anxiety in an elevated plus maze, and anhedonia in the M&M consumption test were more pronounced than those of photon irradiation. Phenylalanine, tyrosine, and tryptophan metabolism, and phenylalanine metabolism and biosynthesis showed very strong pathway changes, following photon irradiation and HU in animals irradiated with 3 Gy. Here, 29 out of 101 plasma metabolites were associated with 1 out of 13 behavioral measures. In the absence of HU, 22 metabolites were related to behavioral and cognitive measures. In HU animals that were sham-irradiated or irradiated with 8 Gy, one metabolite was related to behavioral and cognitive measures. In HU animals irradiated with 3 Gy, six metabolites were related to behavioral and cognitive measures.

**Discussion:** These data suggest that it will be possible to develop stable plasma biomarkers of behavioral and cognitive performance, following environmental challenges like HU and radiation exposure.

## 1 Introduction

Clinical and environmental irradiation can impact the brain function (Raber et al., 2004a; Raber et al., 2004b; Rola et al., 2004; Liu et al., 2010; Braby et al., 2019). There are also concerns that other potential exposures to ionizing radiation via a nuclear accident or terrorist attack could result in behavioral or cognitive changes in people (Braby et al., 2019). Humans are also exposed to substantially lower doses of different types of ionizing radiations in the space environment, and there are concerns that such exposures to the charged-particle environments of space may impact the brain function (Braby et al., 2019). Both galactic cosmic radiation and solar particle events comprise primarily moderate-to-high energy protons, which are largely sparsely ionizing (Townsend et al., 2006). Terrestrial radiation sources that are sparsely ionizing include higher-energy photons produced by X-ray generators and linear accelerators, as well as gamma rays produced by sources such as ^137^Cs or ^60^Co. Whole-body photon irradiation at moderate doses and a high dose rate (1 and 3 Gy; dose rate of 0.69 Gy/min) induces behavioral alterations, including increased measures of anxiety and cognitive injury, and reduces dopamine and GABA levels in 6–8-week-old C57BL6/J male mice 7 days after exposure (Bekal et al., 2021). Post-fear learning photon irradiation (4 Gy; dose rate: 1.25 Gy/min) impairs the extinction of contextual and cued fear memory of 1-month-old C57BL/6J male mice 2 weeks after exposure (Olsen et al., 2014) and enhances cued fear memory of 3-month-old C57BL/6J mice (Olsen et al., 2017). Head-only irradiation of 1.5-month-old Long–Evans male rats with photons (2.3 Gy at a dose rate of 1.9 Gy/min) also causes cognitive injury (Davis et al., 2014). Similarly, whole-brain photon irradiation of 1-month-old Sprague–Dawley rats (20 or 40 Gy; 300 cGy/min) causes cognitive injury at 7 and 20 days post-injury and following exposure to 40-Gy cognitive injury 2 months after exposure (Liu et al., 2010). Head-only irradiation with 27-Gy fractionated exposure of 2.5-month-old Long–Evans rats also impaired cognitive function 5 weeks after radiation exposure (Allen et al., 2018). Whole-brain irradiation of 6-month-old Fischer rats with 25 Gy (single dose) also impairs cognitive function (Akiyama et al., 2001). At doses higher than 5 Gy of photons and 0.5 Gy of ^56^Fe (600 MeV/n), the detrimental effects of irradiation on hippocampal function might involve reduced neurogenesis (Parent et al., 1999; Raber et al., 2004a; Raber et al., 2004b; Rola et al., 2008; Ko et al., 2009; Rosi et al., 2012; Roughton et al., 2012; Rivera et al., 2013; Cacao and Cucinotta, 2016; Sweet et al., 2016; Begolly et al., 2017; Whoolery et al., 2017).

Microgravity is another environmental stressor astronauts experience during space missions. Microgravity affects the brain structure and causes brain dysfunction (Roberts et al., 2019; Hupfeld et al., 2022). Methods devised to simulate microgravity on Earth include hindlimb unloading (HU) (Ray et al., 2001), and brief periods of microgravity have been achieved using parabolic flights (Oman, 2007). Simulated microgravity affects high-level cognition [for a review, see (McNally et al., 2022)]. Simulated microgravity reduces hippocampal levels of pyruvate dehydrogenase, part of glucose metabolism and associates with oxidative stress and brain ischemia, and of the structural protein tubulin (Sarkar et al., 2006) and impairs the 3-dimensional visuospatial tuning and orientation of mice during parabolic flights (Oman, 2007). Irradiation and microgravity might interact in how they affect DNA damage (Moreno-Villanueva et al., 2017) and other injury-related cellular pathways, including oxidative stress, mitochondrial function (Yatagai et al., 2019), cardiovascular health (Patel, 2020), bone function (Krause et al., 2017), and the brain (Raber et al., 2021). Recently, we reported on the complex interaction of simulated microgravity achieved by HU and a simplified field of simulated space radiation (GCRSim) on the behavioral and cognitive performance and metabolic pathways in the plasma and brain of WAG/Rij rats (Raber et al., 2021). Sham-irradiated WAG/Rij rats exposed to simulated microgravity by HU showed impaired hippocampus-dependent spatial habituation learning, but irradiated WAG/Rij rats (1.5 Gy) did not. In addition, rats exposed to 1.5 Gy of GCRSim showed increased depression-like behaviors in the absence but not in the presence of simulated microgravity. Specific behavioral measures such as measures of activity and anxiety and spatial habituation in the open field and depression-like behavior in the forced swim test were associated with plasma levels of distinct metabolites 10 months after behavioral testing. The phenylalanine, tyrosine, and tryptophan metabolism pathway was most profoundly affected; this pathway was affected by radiation in the absence and presence of microgravity in the plasma and by microgravity by itself.

To determine whether these effects of irradiation and simulated microgravity are specific to WAG/Rij rats and whether these effects are specific to simulated space radiation, we characterized the behavioral and cognitive performance of male Fischer rats 2 months after sham irradiation or total body irradiation with photons (3, 8, or 10 Gy) in the absence or presence of simulated microgravity delivered by HU. These radiation doses were based on values used in previous rat studies that determined a relative biological effectiveness for perivascular cardiac fibrosis, defined as an increase in the perivascular cardiac collagen content, in the setting of deep space exploration. The relative biologic effectiveness represents the ratio of doses required by two different irradiation types (e.g., photons and galactic cosmic radiation) to cause the same level of biologic effect. In a previous study in WAG/RijCmcr rats, the increase in perivascular collagen occurred after a much lower total dose of 1.5 Gy from a three-ion beam grouping, representative of galactic cosmic radiation, as compared with 10 Gy of photon irradiation (Lenarczyk et al., 2023). The range of radiation doses in the present study was expanded to include 3, 8, and 10 Gy. The 3-Gy dose was selected as previous studies have shown that behavioral performance can be affected at this low dose (Bekal et al., 2021). In addition, we analyzed the plasma collected 9 months after sham irradiation or total body irradiation for distinct alterations in metabolic pathways and to determine whether changes to metabolic measures were associated with specific behavioral and cognitive measures. The results from the present study may be used to determine the relative biological effectiveness for behavioral performance and metabolic pathways in future studies for Fischer rats to compare our findings with behaviorally tested WAG/RijCmcr rats.

## 2 Materials and methods

### 2.1 Animals and radiation exposures

**FIGURE 1:**
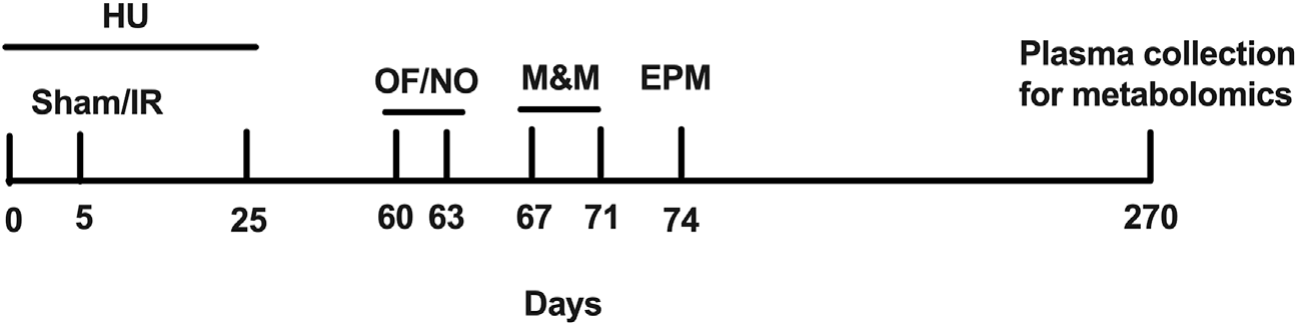
Experimental design of the study. Sham, sham irradiation; IR, irradiation; OF, open field; NO, novel object; M&M, M&M test; EPM, elevated plus maze.

In this study, 344 7–8-month-old male Fischer rats (RRID:RGD_734,478) (*n* = 97) were shipped to the Wake Forest University School of Medicine, Winston-Salem, NC (see Figure 1 for the experimental design of this study). Rats were selected for this study because previous studies have shown that whole-body exposure to low-linear energy transfer radiation results in the development of cardiovascular disease in a time- and dose-dependent manner. In previous studies, WAG/RijCmcr rats were used as they are an established model of sensitivity to radiation injury in the heart, following exposure to photons. To investigate whether another strain of rats would be either sensitive or resistant to ionizing radiation and, thus, suitable for future flight studies aboard the International Space Station, we selected the Fischer rat. This strain of rat, unlike the WAG/RijCmcr rat, does not continue to gain weight as an adult and, so, is better suited to be housed in the Rodent Research Hardware System in use aboard the International Space Station. At 9 months of age, they were sham-irradiated or irradiated with photons (3, 8, or 10 Gy). Experimental groups of simulated weightlessness using HU alone and HU in combination with photon irradiation were included as part of the paradigm. The HU procedure was performed 5 days prior to sham irradiation or irradiation, as described below. The rats remained under the HU condition, 25 days after sham irradiation or irradiation. One week after the HU period, the animals were shipped to the Medical College of Wisconsin (MCW). The animals were maintained on a Teklad 8904 diet (Indianapolis, IN) and fed *ad libitum* during this study. The animals were housed in a reverse-light cycle room with lights off from 07.30 to 19.30. All behavioral and cognitive testing was performed during the dark period, starting 2 months after irradiation or sham irradiation and over 1 month after the HU procedure. During deep-space exploratory missions, crew members are exposed to space radiation and microgravity for prolonged periods. In a previous study, we characterized behavioral and cognitive performance of WAG/Rij rats 3 months after sham irradiation or total body irradiation with a simplified 5-ion beam representative of GCR in the absence or presence of HU (Raber et al., 2021). To determine whether behavioral performance would be impacted by these spaceflight stressors at an earlier time point, we selected a 2-month period after total body irradiation with photons in the absence or presence of HU before measurements in Fischer rats were made. As the animals received HU 25 days following sham irradiation and irradiation, this provided the animals time to recover from the HU period. All animal procedures were consistent with ARRIVE guidelines and reviewed and approved by the Institutional Animal Care and Use Committee (IACUC) at the Wake Forest University School of Medicine and MCW. All analyses were performed blinded to treatment. The code was broken once the data were analyzed.

### 2.2 HU

On day 1, the rats were randomly grouped to serve as either full weight-bearing (non-HU) or to be HU via tail suspension. The HU procedure was performed the same way, as previously published (Wiley et al., 2016; Raber et al., 2021). Briefly, the rats were lightly anesthetized with isoflurane (2.5%–3.0%; 5% oxygen flow rate). Benzoin tincture was applied to the cleaned, lateral surfaces of the tail before placing a free end of the adhesive medical traction tape (1-cm width, 30-cm length, 3M, St. Paul, MN, United States) at approximately 1 cm from the base of the tail and then adhered to 3/4th of the length of the tail. The free end of the tape was looped through a catch on a custom plastic ball-bearing swivel and symmetrically adhered to the other lateral side of the tail. Half-inch micropore tape (3M, St. Paul, MN, United States) strips were then secured perpendicularly over the traction tape. A 1.56-inch carabiner clip (Nite Ize, Boulder, CO, United States) was attached to both the swivel and a zipper hook that could be adjusted vertically along an attached perlon cord (STAS Group, BA, Eindhoven, Netherlands). The cord was attached to a pulley (Blue Hawk, Gilbert, AZ, United States) that permitted the entire setup to slide securely over a 5/16-inch-diameter round, solid steel rod (Hillman, Cincinnati, OH, United States). The adjustment of the zipper lock along the Perlon cord lifted the hind limbs fully off the substrate, with the abdomen and thorax at a 30-degree horizontal form. Sham HU rats received isoflurane but were not tail-suspended. All rats were singly housed, assigned to receive one of the three radiation doses (3, 8, or 10 Gy) or sham irradiation (0 Gy). Wet chow was provided to ensure hydration.

### 2.3 Photon irradiation

All radiation dosimetry procedures were performed and validated as per previous studies (Walb et al., 2015; Willey et al., 2016). The animals were placed on the treatment couch of an Elekta 6-MV photon linear accelerator (Elekta Synergy, Elekta AB, Stockholm, Sweden). Consistent with our earlier radiation study (Wiley et al., 2016; Raber et al., 2021), doses were delivered in two fractions, using laterally opposed beams at 0.5 Gy/min. For both HU and sham HU groups, 4 cm of homogenous brass shielding was placed between the hind limbs from the beam to permit the repopulation of hematopoietic stem cells; dosimetry was performed with shielding in place. Following irradiation procedures, the rats were immediately returned to normal housing conditions (HU or full weight-bearing).

### 2.4 Performance in the open field in the absence and presence of objects (week 1)

Exploratory behavior, measures of anxiety, and hippocampus-dependent spatial habituation learning were assessed in a black open field (90.49 cm × 90.49 cm × 45.72 cm) for 2 subsequent days, as in an earlier study with middle-aged, male WAG/Rij rats (Raber et al., 2021). For open-field testing, each experimental group had 10 rats/group, with the exception of the non-HU 8-Gy and HU 8-Gy groups, which had 9 rats/group, for a total of 78 rats. The animal cage was brought into the testing room. The rat was picked up by gently placing our thumb and forefinger behind the forelegs and wrapping the hand around the stomach. The researcher held the animal against the stomach while transporting the animal to or from the enclosure. The animal was placed in the middle of the open field for 5 min per day. The researcher left the testing room during behavioral testing. Following testing of each rat, the enclosure was cleaned with a 70% isopropyl alcohol solution. The outcome measures analyzed using video tracking (ANY-maze software, Stoelting Co., Wood Dale, IL, United States) were the total distance moved, the time spent freezing, and the percentage of time spent in the more anxiety-provoking center zone (45.25 × 45.25 cm). Freezing is defined as the lack of movement besides respiration.

On day 3, the rats were tested in the open field containing objects based on the protocol using 3D-printed objects, as reported previously, with the following modifications (Aguilar et al., 2018). The objects were placed 45.72 cm from the top side of the enclosure and 30.48 cm from the left and right sides of the enclosure, with the distance between the two objects being also 30.48 cm. The rats were placed back in the enclosure containing 2 identical blue objects (squares) or 2 identical red objects (cylinders) for an acquisition trial of 5 min. The objects were counterbalanced for this task. Half the rats started with the blue squares, and the other half started with the red cylinders. Then, 60 min following the acquisition trial (A), the rats were placed back in the enclosure with one of the familiar objects replaced with a novel red or blue object for a test trial (T). The outcome measures analyzed using video tracking were the total distance moved and percentage of time spent in the center zone (45.25 × 45.25 cm) containing the objects. In addition, the discrimination index, defined as the time spent exploring the novel object minus the time spent with the familiar object divided by the time spent exploring both objects, was analyzed by manual scoring of the digital videos. The light intensity during open-field and object recognition testing was 50 lux. A white noise generator (setting II) and overhead lights were used during the testing.

### 2.5 Anhedonia test (week 2)

In the week following open-field and object recognition testing, anhedonia of the rats was tested using the M&M test (Bolton et al., 2018). Chocolate is safe for rats in small amounts (Carpanini et al., 1978; Tarka Jr et al., 1991). The main ingredient in chocolate that causes issues in cats and dogs is theobromine (Carpanini et al., 1978), but rats can process this chemical as humans do. We recognize that it is possible to administer too much chocolate to a rat, but this would require the equivalent of a 150-g chocolate bar. In our study, we only provided 10 g of M&M for 1 h at a time, and there were maybe one or two incidents where we provided an additional 2 g. The symptoms of theobromine poisoning include diarrhea and increased urination. We did not observe any of these symptoms in the rats. Other than theobromine poisoning, long-term chocolate consumption also affects mortality (Sun et al., 2022). Considering that we only provide chocolate access for 1 h every 3 days, it cannot be considered for a long period. On day 1, each rat received 2 M&Ms (multi-colored button-shaped chocolates, Mars Inc., McLean, VA, United States) in the home cage. For anhedonia testing, all experimental groups had 12 rats/group, with the exception of the non-HU 3-Gy group and the HU 0 (*n* = 11 rats)- and 3-Gy groups (*n* = 13 rats), for a total of 97 rats. Consumption was assessed after 10 min by weighing the remaining M&Ms. On days 2–4, the rats were moved into a testing room, separate from the housing room. Each rat was placed in a clean cage without bedding (no available food or water) and allowed to habituate for 1 h. A pre-weighed weighing boat containing 10 g of M&Ms was placed in the center of the cage. The weighing boat was attached to the bottom of the cage using a tape. The M&M-filled weighing boat was left in the cage for up to 1 h. If the weighing boat was empty before the end of the hour, a new weighing boat with M&Ms was placed in the cage. After 1 h, the animal was placed back in its home cage. The weighing boat was removed from the testing cage. Any M&M crumbs were removed from the cage and placed in the weighing boat if they were not soiled by rat feces. The weighing boat containing M&Ms was weighed again to calculate M&M consumption for that day. The average consumption of M&Ms over the 3 days of testing and the percentage of M&Ms consumed on days 2 and 3 compared to day 1 were calculated.

### 2.6 Elevated plus maze (week 3)

Measures of anxiety-like behavior were assessed in the elevated plus maze. The arms were 50-cm long, 10-cm wide, and 40-cm high. The maze was positioned 50 cm above the floor. The duration of the test was 5 min. For elevated plus maze testing, as for open-field testing, all experimental groups had 10 rats/group, with the exception of the non-HU 8-Gy and HU 8-Gy groups, which had 9 rats/group, for a total of 78 rats. The following distinct outcomes were measured using ANY-maze video tracking software: 1) ratios of the time spent in the open areas (defined as time spent in the open arms divided by time spent in the open + closed arms); 2) ratios of the entries in the open arms (entries into the open areas divided by entries into the open + closed arms); 3) distance moved in the open and closed arms; 4) time spent mobile; 5) time spent freezing; and 6) time spent in the intersection.

### 2.7 Metabolomics analysis of plasma

Nine months following sham irradiation or irradiation and 7 months after the behavioral and cognitive testing, the blood of 72 rats (9 rats/experimental group) was collected in EDTA-containing tubes and centrifuged at 2,000 g for 10 min, and the plasma supernatant was collected and stored at −80°C for plasma metabolomics. Degenerative, radiation-induced cardiovascular disease can follow exposure to conventional photon irradiation (e.g., following breast radiotherapy) and takes many years to develop. Thus, the increased cardiac risk to astronauts would be expected to remain well after they return to Earth, and this is reflected in the design of rat studies of radiation-induced cardiovascular disease. We used a similar experimental timeline for metabolic pathway studies in rats that results in radiation-induced cardiovascular disease. These longer experimental timelines are used to determine whether humans exposed to lower, mission-relevant doses of galactic cosmic radiation develop radiation-induced heart disease and changes in metabolic pathways. Metabolites were extracted from 100 μL of plasma. Untargeted metabolomics analysis was performed as described (Kirkwood et al., 2013). Liquid chromatography (LC) was performed using a Shimadzu Nexera system with an Inertsil Phenyl-3 Column (4.6 × 150 mm, 100 Å, 5 μm; GL Sciences, Rolling Hills Estates, CA, United States) coupled to a quadrupole time-of-flight (Q-TOF) mass spectrometer (AB SCIEX, TripleTOF 5600) operated in the information-dependent MS/MS acquisition mode. The samples were ordered randomly, and multiple quality control samples were included. QC samples were generated by pooling 10-µL aliquots from the plasma sample extracts and analyzed along with the samples. All samples were run in the positive and negative ion modes. In case metabolites were present in both ion modes, the mode with the higher peak value was selected for further analysis. The column temperature was held at 50 °C, and the samples were maintained at 10 °C. The metabolomics data were processed using MarkerView (SCIEX, Framingham, MA) and PeakView (SCIEX, Framingham, MA) software programs for integrated pathway and statistical analyses, respectively. The identification of metabolites was based on mass error (<30 ppm) and MS/MS fragment ions. The metabolites were also confirmed using the retention time and mass-to-charge (m/z) ratio, and by comparing to authentic standards (±1 min) from an in-house library (IROA Technologies, Bolton, MA), allowing for the streamlined identification of metabolites. LIPID MAPS (Welcome Trust, United Kingdom), METLIN (Scripps, La Jolla, CA), and the Human Metabolome Database (HMDB) (University of Alberta, Edmonton, Canada) were used for MS and MS/MS matching. MetaboAnalyst pathway analysis (Montreal, Quebec, Canada) was performed, as described by Xia and Wishart (2010) and Kirkwood et al. (2013). Raw metabolite peak values in the plasma were analyzed without log transformation or Pareto scaling. Four distinct comparative analyses were performed: 1) effects of radiation, for each dose compared to sham irradiation, in rats without HU; 2) effects of radiation, for each dose compared to sham irradiation, in rats with HU; 3) effects of HU in sham-irradiated rats; and 4) effects of HU in irradiated rats, for each dose compared to sham irradiation. The pathways were visualized using scatter plots (testing significant features) in MetaboAnalyst, with the “global test” and “relative-betweenness centrality” as parameters for the enrichment method and topological analysis, respectively. In case pathways were revealed to be significantly affected, we used box and whisker plots to indicate the direction of change in the affected metabolites in those affected pathways.

### 2.8 Statistical analyses

All data are presented as the mean ± standard error of the mean (SEM). Behavioral and cognitive data were analyzing using SPSS v.25 software (IBM, Armonk, NY, United States) and R statistical programming language v.4.3.1. To analyze the behavioral and cognitive performance after sham irradiation or photon irradiation (0, 3, 8, or 10 Gy), we performed analysis of variance (ANOVA) with Dunnett’s *post hoc* tests compared to one control group. To assess the role of microgravity, we performed ANOVA with radiation (0, 3, 8, or 10 Gy) and HU condition (control or HU) as between-group factors with repeated measures when appropriate. For some analyses, as indicated and appropriate, two-sided t-tests were used. We set statistical significance to *p* < 0.05. Greenhouse–Geisser corrections were used if sphericity was shown to be violated (Mauchly’s test).

Metabolomics in plasma were analyzed for the comparisons described above. To identify potential plasma biomarkers of radiation exposure or HU conditions in behavioral or cognitive performance, we used univariate linear regression analyses stratified by radiation exposure and the HU condition, with Z-scores for behavioral measures as the dependent variable and metabolite values as the independent variable. We selected those metabolites that were most consistently included in the models and those that were most consistently associated with an outcome (*p* < 0.05) in the models (at least four times as there were four activity measures and four anxiety measures: activity in the open field with and without objects in 4 subsequent trials, time spent in the center of the open field containing objects, and time spent exploring the objects in two trials); we selected and limited the analysis to 13 behavioral measures to reduce the risk of a type I error. We added an anxiety measure in the elevated plus maze and a depression-like measure (anhedonia) in the current study. Both tests were not included in the previous study. Table 1 lists all behavioral measures used for the regression analysis in the current study, and the footnote describes the comparison of this list with that of our earlier study.

**TABLE 1:** Behavioral measures included for the regression analysis with individual metabolites^a^.

Measure	Definition
Anhedonia	Mean percentage change in consumption of days 2 and 3 compared to day 1.
Activity	Distance moved on day 1 of the open-field test.
Activity	Distance moved on day 2 of the open-field test.
Activity	Distance moved in the object-recognition training trial.
Activity	Distance moved in the object-recognition test trial.
Anxiety	Percentage of time spent in the center of the open field on day 1 of open-field testing.
Anxiety	Percentage of time spent in the center of the open field on day 1 of open-field testing.
Anxiety	Ratio times spent in the open arms/open + closed arms.
Cognition	Total duration spent exploring the objects during the training trial.
Cognition	Total duration spent exploring the objects during the test trial.
Cognition	Percentage of time spent in the center of the open field during the object-recognition training trial.
Cognition	Percentage of time spent in the center of the open field during the object-recognition test trial.
Cognition	Discrimination index in the object-recognition test.

^a^ The same measures were used in the current study and our earlier study in WAG/Rij rats with the following exceptions: 1) in the current study, a measure of anxiety in the elevated plus maze was included. The elevated plus maze was not included in the earlier study; 2) in the current study, an anhedonia test was used instead of the forced swim test that was included in the earlier study; and 3) in the current study, there were two trials in the object recognition test: a training trial and a testing trial; in the previous study, there were three trials: habituation, acquisition, and testing trials.

MetaboAnalyst software was used to generate impact plots. Graphs were generated using GraphPad software v.8.2.0 (La Jolla, CA, United States). As MetaboAnalyst is not ideal to analyze amino acid-related pathways, we analyzed them separately as described under Metabolomics Analysis above.

## 3 Results

### 3.1 Effects of HU and radiation on the performance in the open field without and with objects

**FIGURE 2:**
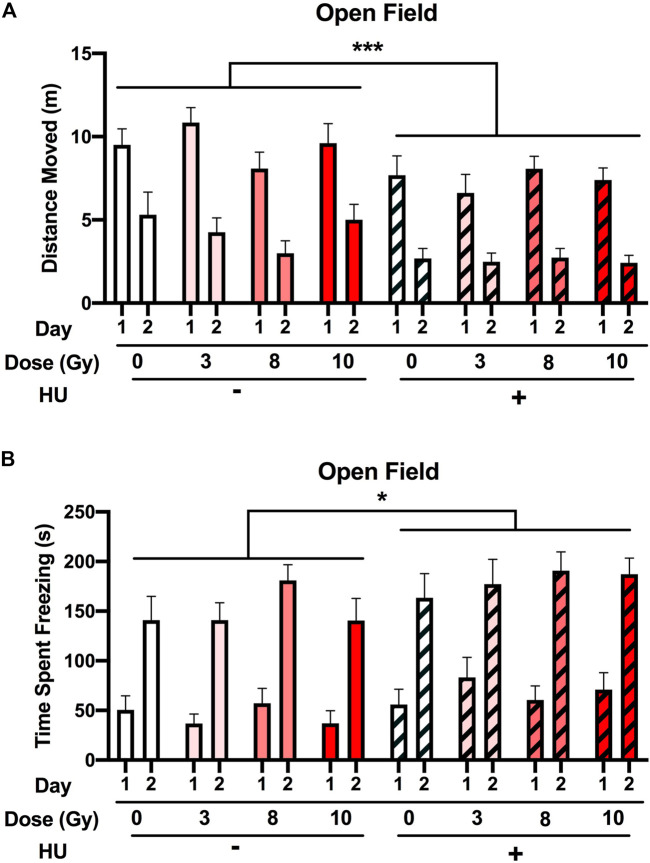
Performance in the open field in the absence of objects. **(A)** The rats were tested for exploratory behavior in the open field on 2 subsequent days. The trials last 5 min and were conducted 24 h apart. **(A)** HU-treated animals moved less than non-HU animals. ***p < 0.001. An effect of day was observed in all groups showing spatial habitation learning and moving less on day 2 than on day 1. **(B)** Freezing levels were higher in HU animals than in non-HU animals. *p < 0.05. An effect of day was observed in all groups freezing more on day 2 than on day 1. No effect of radiation was observed on activity or freezing levels in the open field.

When activity levels were analyzed for 2 subsequent days in the open field, an effect of HU was observed (*F* (1, 89) = 14.476, *p* < 0.001), with HU-treated animals moving less than non-HU animals (Figure 2A). An effect of the day was also observed, with all groups showing spatial habitation learning and moving less on day 2 than on day 1 [*F* (1, 89) = 174.910, *p* < 0.001]. In addition, HU affected the freezing levels in the open field [*F* (1, 89) = 5.106, *p* = 0.026], with higher freezing levels observed in HU animals than in non-HU animals (Figure 2B). An effect of the day was also observed, with all groups freezing more on day 2 than on day 1 [*F* (1, 89) = 313.051, *p* < 0.001]. No effect of radiation was observed on the activity or freezing levels in the open field.

Next, we assessed the time spent in the more anxiety-provoking center of the open field. HU [*F* (1, 89) = 0.656, *p* = 0.420] or radiation [*F* (3, 89) = 0.418, *p* = 0.741] did not affect the time spent in the center of the open field (Supplementary Figure S1).

**FIGURE 3:**
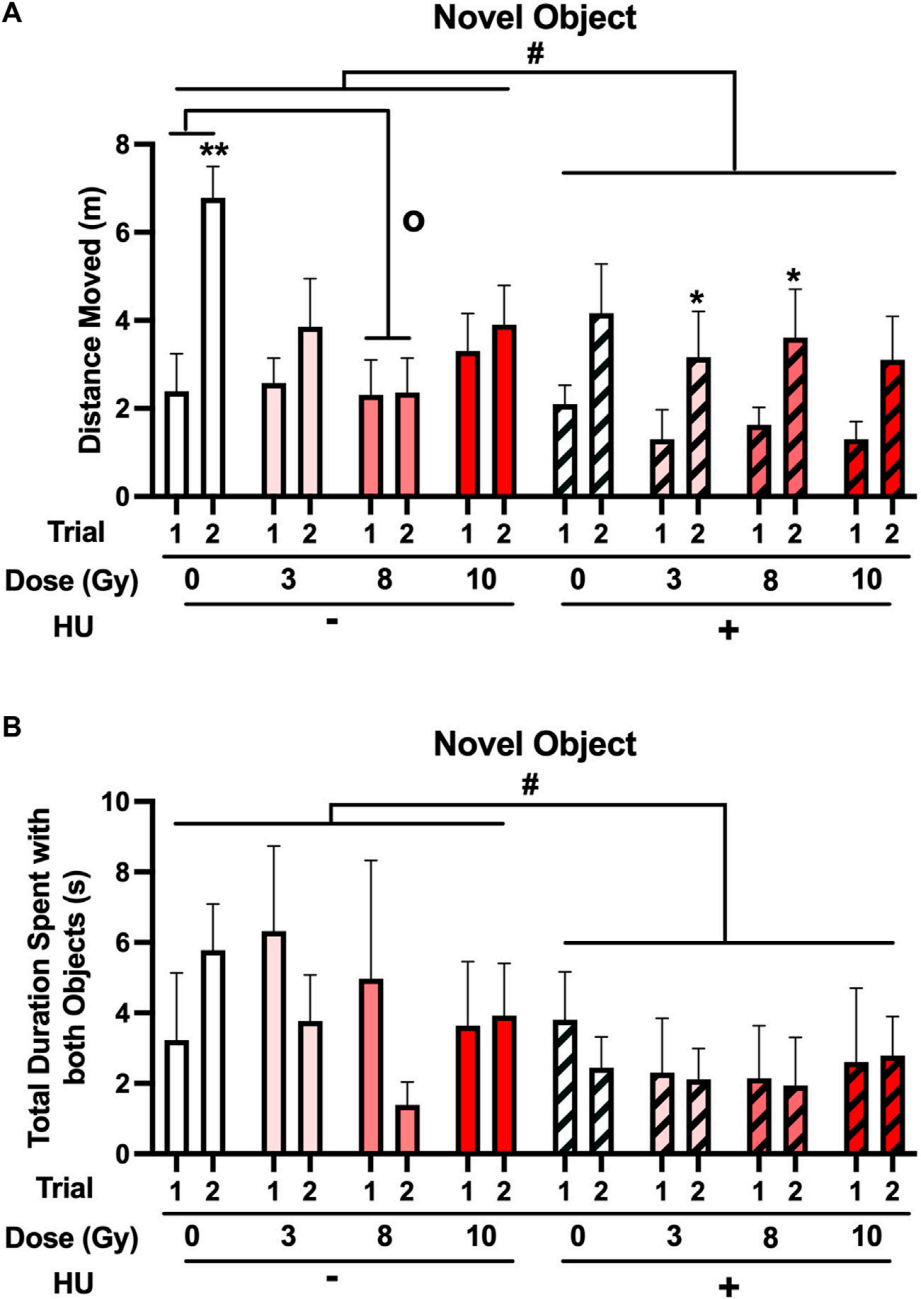
**(A)** When the performance in the open field containing objects was assessed, a trend toward an effect of HU on activity levels was observed, with lower activity levels in HU animals than in non-HU animals. ^#^ *p* = 0.072). In the non-HU group, a radiation × trial interaction [*F* (3, 70) = 5.991, *p* = 0.002] and an effect of trial [*F* (1, 35) = 15.818, *p* < 0.001] were observed. The animals irradiated with 8 Gy moved less than the sham-irradiated animals (^o^ *p* < 0.05, Dunnett’s test). In addition, under the non-HU condition, sham-irradiated rats moved more in trial 2 than in trial 1. **p = 0.001. Under the HU condition, animals irradiated with 3 or 8 Gy moved more in trial 2 than in trial 1. *p < 0.05. **(B)** A trend toward the effect of HU on time spent exploring the objects was observed, with HU animals spending less time spent exploring the objects than non-HU animals. ^#^ *p* = 0.069.

Next, the performance in the open field containing objects was assessed. No significant effect of HU was observed on activity levels [*F* (1, 70) = 3.335, *p* = 0.072] (Figure 3A) or a significant radiation × trial interaction [*F* (3, 70) = 2.434, *p* = 0.072]. When the non-HU group was analyzed, a radiation × trial interaction [*F* (3, 70) = 5.991, *p* = 0.002] and an effect of day [*F* (1, 35) = 15.818, *p* < 0.001] were observed. Although there was no overall effect of radiation under the non-HU condition, the animals irradiated with 8 Gy moved less than the sham-irradiated animals (*p* = 0.0255, Dunnett’s test). In addition, under the non-HU condition, only sham-irradiated rats moved more in trial 2 than in trial 1 (*p* = 0.001). In contrast, under the HU condition, only an effect of the trial (*F* (1, 35) = 14.006, *p* = 0.001) was observed, and animals irradiated with 3 (*p* = 0.044) or 8 (*p* = 0.038) Gy moved more in trial 2 than in trial 1.

No significant effect of HU was observed on the time spent exploring the objects, with HU animals spending less time spent exploring the objects than non-HU animals [*F* (1, 70) = 3.412, *p* = 0.069] (Figure 3B).

We also analyzed the time spent in the center of the open field containing the objects. No significant effect of HU [*F* (1, 69) = 3.160, *p* = 0.080] or radiation [*F* (3, 69) = 1.118, *p* = 0.348] was observed.

Finally, we analyzed the percentage of time spent with familiar and novel objects. As the animals spent very little time exploring the objects and 38% of the animals did not explore the objects at all in the test with the novel object, object recognition could not be reliably assessed. None of the groups had a positive discrimination index significantly different from 0.

### 3.2 Anhedonia test

**FIGURE 4:**
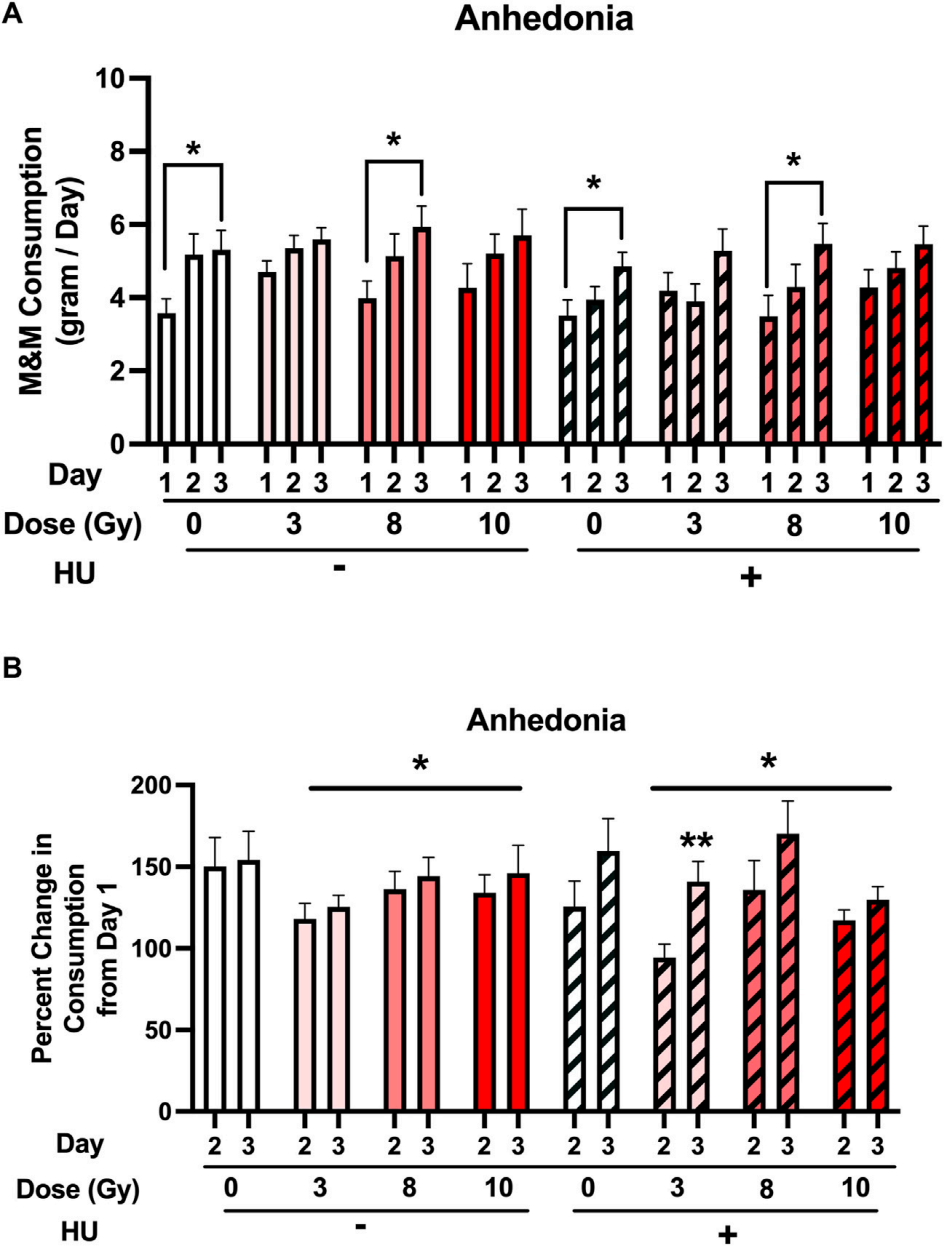
**(A)** M&M consumption over the 3 days of the test was analyzed. An HU × day interaction [*F* (2, 178) = 3.944, *p* = 0.021] and an effect of day [*F* (2, 178) = 55.933, *p* < 0.001] were observed. A trend toward an effect of HU was observed, but it did not reach significance (*p* = 0.099). Under the non-HU condition, sham-irradiated rats showed more M&M consumption on day 3 than on day 1. A trend toward more M&M consumption on day 2 than on day 1 was observed, but it did not reach significance (*p* = 0.057). Under the HU condition, sham-irradiated rats and those irradiated with 8 Gy showed more M&M consumption on day 3 than on day 1. *p < 0.05. **(B)** Percentage of consumption on days 2 and 3 compared to day 1. An effect of radiation was observed, with a lower percentage of M&M consumption in irradiated rats than in sham-irradiated rats. *p < 0.05 *versus* sham irradiation. An effect of day [*F* (1, 86) = 14.041, *p* < 0.001] and a HU × day interaction [*F* (1, 86) = 5.133, *p* = 0.026] were observed. Under the non-HU condition, a trend toward a lower percentage of consumption was observed in animals irradiated with 3 Gy than in sham-irradiated rats, but it did not reach significance (*p* = 0.0521). Under the HU condition, in rats irradiated with 3 Gy, the percentage of M&M consumption on day 3 was higher than that on day 2. **p = 0.006. A trend toward a higher percentage of M&M consumption was observed on day 3 than on day 2 in rats irradiated with 8 Gy, but it did not reach significance (*p* = 0.059).

First, the M&M consumption over the 3 days of the test was analyzed. An HU × day interaction [*F* (2, 178) = 3.944, *p* = 0.021] and an effect of day [*F* (2, 178) = 55.933, *p* < 0.001] were observed (Figure 4A). No significant effect of HU [*F* (1, 89) = 2.777, *p* = 0.099] was observed. Under the non-HU condition, sham-irradiated rats consumed more M&Ms on day 3 than on day 1 (*p* = 0.0367). No significant change was observed in M&M consumption on day 2 compared to day 1 (*p* = 0.057). Under the non-HU condition, the rats irradiated with 8 Gy consumed more M&Ms on day 3 than on day 1 (*p* = 0.0336). No difference in M&M consumption between days 1 and 3 was observed in rats irradiated with 3 or 10 Gy. Under the HU condition, sham-irradiated rats (*p* = 0.0396) and those irradiated with 8 Gy (*p* = 0.0421) consumed more M&Ms on day 3 than on day 1. Like in the non-HU condition, no difference in M&M consumption between days 1 and 3 was observed in rats irradiated with 3 or 10 Gy under the HU condition.

Next, the percentage of consumption on days 2 and 3 compared to day 1 was analyzed. An effect of radiation [*F* (3, 86) = 2.779, *p* = 0.046] was observed, with a lower percentage of M&M consumption in irradiated rats than in sham-irradiated rats (Figure 4B). An effect of day [*F* (1, 86) = 14.041, *p* < 0.001] and a HU × day interaction [*F* (1, 86) = 5.133, *p* = 0.026] were observed. Under the non-HU condition, no significant lower percentage of consumption was observed in animals irradiated with 3 Gy than in sham-irradiated rats (*p* = 0.0521). Under the HU condition, in rats irradiated with 3 Gy, the percentage of M&M consumption on day 3 was higher than that on day 2 (*p* = 0.006), and there was a trend toward a higher percentage of M&M consumption on day 3 than on day 2 in rats irradiated with 8 Gy, but it did not reach significance (*p* = 0.059).

### 3.3 Elevated plus maze

**FIGURE 5:**
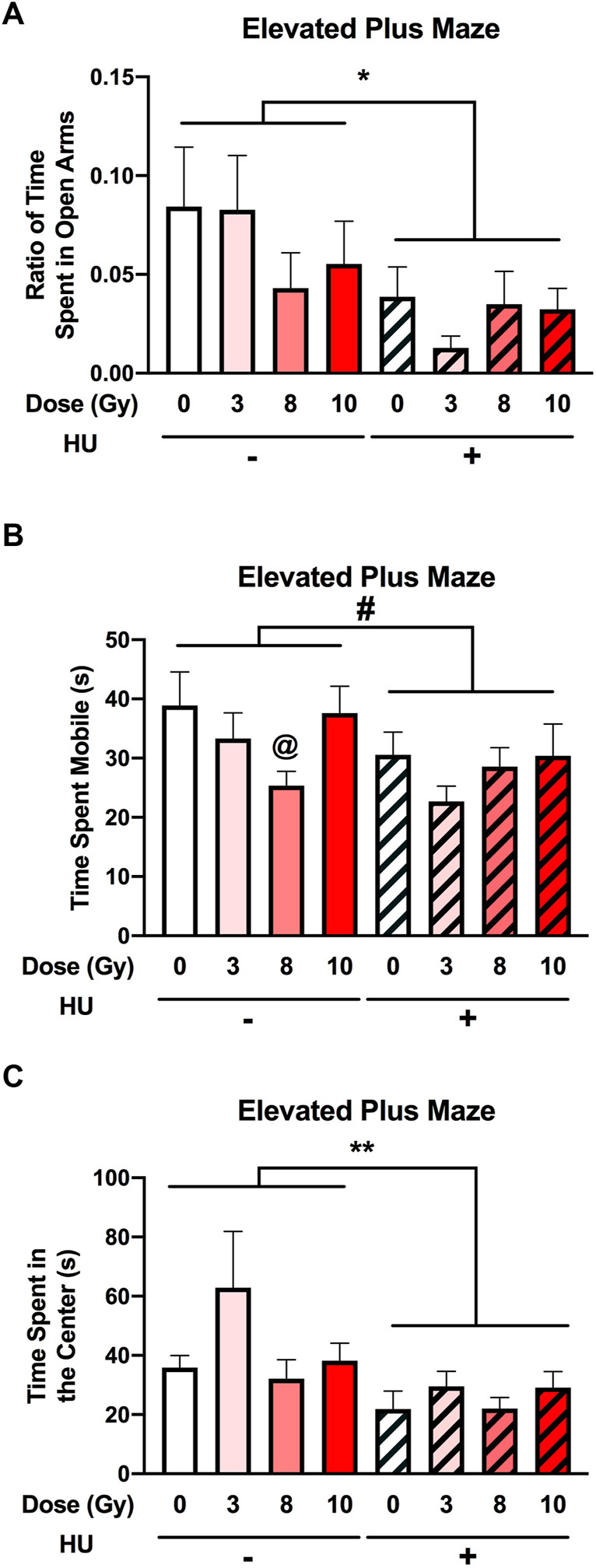
Measures of anxiety in the elevated plus maze. **(A)** The ratio of time spent in the open arms was lower in HU animals than in non-HU animals. *p < 0.05. **(B)** A trend toward HU animals spending less time mobile than non-HU animals was observed. ^#^ *p* = 0.05. Under the non-HU condition, a trend toward rats irradiated with 8 Gy spending less time mobile than sham-irradiated rats was observed. ^@^ *p* = 0.093. **(C)** HU animals spent less time in the center of the elevated plus maze than non-HU animals. **p = 0.009.

An effect of HU on the measures of anxiety in the elevated plus maze was observed. The ratio of the time spent in the open arms was lower in HU rats than in non-HU animals [*F* (1, 89) = 6.811, *p* = 0.011] (Figure 5A). The ratio entry measure was not affected by HU or radiation (Supplementary Figure S2).

No significant effect of HU was observed on activity levels in the elevated plus maze [*F* (1, 89) = 3.824, *p* = 0.054] (Figure 5B). Under the non-HU condition, the rats irradiated with 8 Gy did not spend significantly less time mobile than sham-irradiated rats (*p* = 0.093).

An effect of HU on the time spent in the center of the elevated plus maze [*F* (1, 89) = 7.228, *p* = 0.009] was observed, with HU animals spending less time in the center than non-HU animals (Figure 5C).

### 3.4 Metabolomics analysis of plasma

**FIGURE 6:**
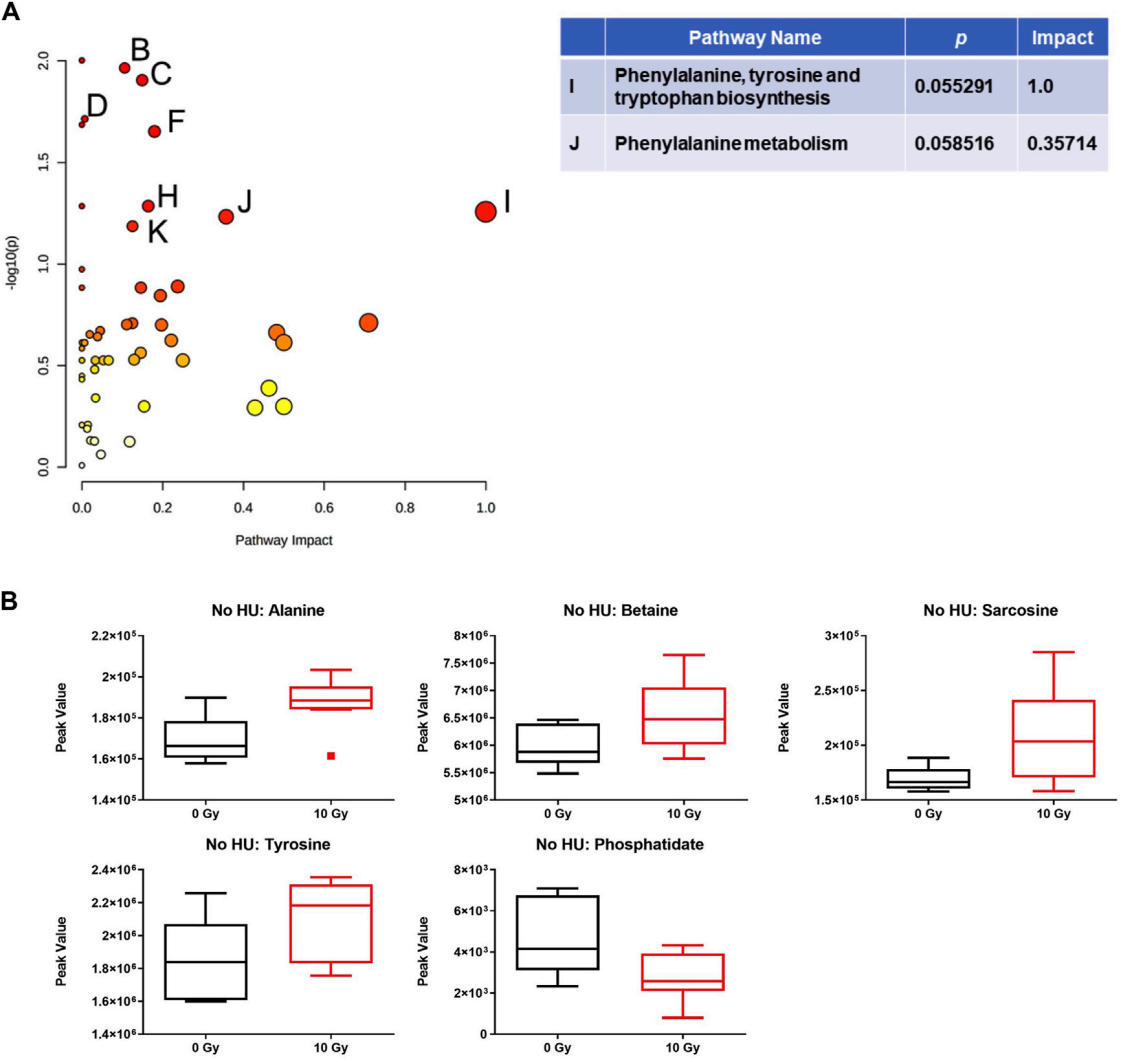
Effects of radiation on metabolic pathways in the plasma in the absence of HU. **(A)** Analysis of the effects of 10-Gy radiation in the plasma in the absence of HU showed that the phenylalanine, tyrosine, and tryptophan biosynthesis pathway was affected. Affected pathways with a lower pathway impact were *B)* glycerolipid metabolism, *p*-value: 0.010842; *C)* glycerophospholipid metabolism, *p*-value: 0.012451; *D)* pantothenate and CoA biosynthesis, *p*-value: 0.019314; *E)*: beta-alanine metabolism, *p*-value: 0.02063; *F*: glycine, serine, and threonine metabolism, *p*-value: 0.022268; and two pathways with borderline significance; *H)*: tyrosine metabolism, *p*-value: 0.051856; and *K)*: cysteine and methionine metabolism, *p*-value: 0.06513. **(B)** With the exception of phosphatidate, the plasma metabolite levels were increased by 10-Gy radiation. The metabolome view figures contain all the matched pathways (the metabolome) arranged by *p-*values (generated as part of the pathway enrichment analysis) on the Y-axis and pathway impact values (generated as part of the pathway topology analysis) on the X-axis. The node color is based on its *p*-value, and the node radius is determined based on their pathway impact values.

Analysis of the effects of radiation in the plasma in the absence of HU showed that the phenylalanine, tyrosine, and tryptophan biosynthesis pathway was affected, comparing sham irradiation *versus* irradiation with 10 Gy (Figure 6A). With the exception of phosphatidate, the plasma metabolite levels were increased by 10-Gy radiation (Figure 6B).

**FIGURE 7:**
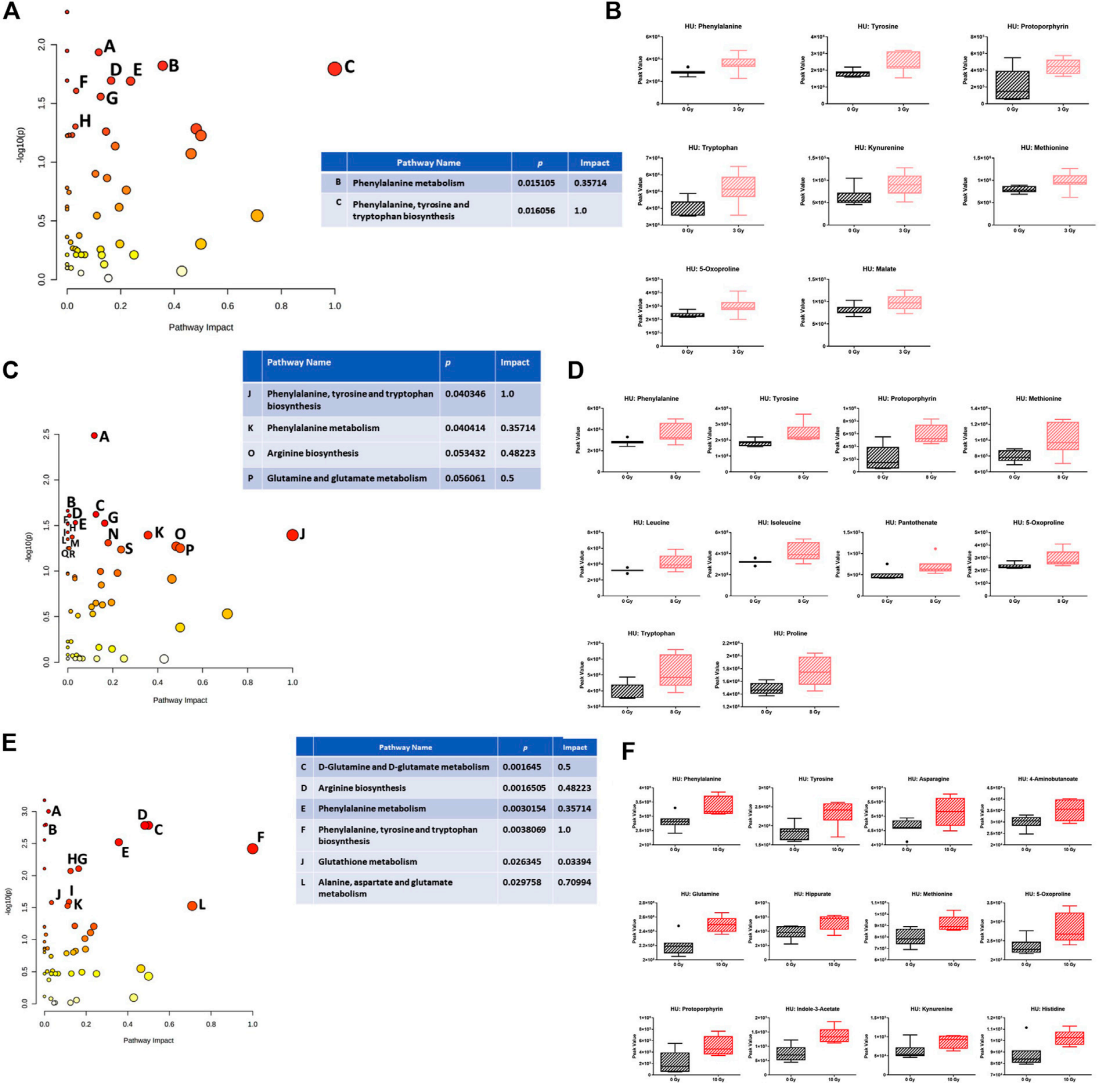
**(A)** Effects of radiation on metabolic pathways in the plasma in the presence of HU. Within the HU groups, comparing sham irradiation *versus* 3-Gy irradiation, the phenylalanine, tyrosine, and tryptophan biosynthesis pathways were affected. Affected pathways with a lower pathway impact were *A)* porphyrin and chlorophyll metabolism, *p*-value 0.011605; *D)* tyrosine metabolism, *p*-value 0.020236; *E)* tryptophan metabolism, *p*-value 0.020423; *F)* glutathione metabolism, *p*-value 0.024727; *G)* cysteine and methionine metabolism, *p*-value 0.027695; and *H)* pyruvate metabolism, *p*-value 0.049762. **(B)** Plasma levels of the individual metabolites were increased, following irradiation. **(C)** Within the HU groups, comparing sham irradiation *versus* 8-Gy irradiation, the phenylalanine, tyrosine, and tryptophan biosynthesis pathways were also affected. The arginine biosynthesis and glutamine and glutamate metabolism pathways were also affected but with a much lower impact. Affected pathways with a lower pathway impact were *A)* porphyrin and chlorophyll metabolism, *p*-value 0.0032677; *B)* valine, leucine, and isoleucine biosynthesis, *p*-value 0.021871; *C)* cysteine and methionine metabolism, *p*-value 0.023914; *D)* pantothenate and CoA biosynthesis, *p*-value 0.024702; *E)* glutathione metabolism, *p*-value 0.029465; *F)* ubiquinone and other terpenoid–quinone biosynthesis, *p*-value 0.02985; *G)* tyrosine metabolism, *p*-value 0.02985; *H)* aminoacyl–tRNA biosynthesis, *p*-value 0.030508; *I)* steroid biosynthesis, *p*-value 0.037586; *L)* purine metabolism, *p*-value 0.042312; *M)* beta-alanine metabolism, *p*-value 0.044738; and *N)* glycine, serine, and threonine metabolism, *p*-value 0.049054. **(D)** The individual metabolites in the pathway were also increased, following irradiation. **(E)** Within the HU groups, comparing sham irradiation *versus* 10-Gy irradiation, the phenylalanine, tyrosine, and tryptophan biosynthesis pathways were also most clearly affected. Glutamine and glutamate metabolism, arginine biosynthesis, phenylalanine metabolism, glutathione metabolism, and alanine, aspartate, and glutamate metabolism pathways were also affected but with a lower pathway impact. Affected pathways with a lower pathway impact were *A)* purine metabolism, *p*-value 9.9526E-4; *B)* pyrimidine metabolism, *p*-value 0.0016094; *G)* tyrosine metabolism, *p*-value 0.0077996; *H)* cysteine and methionine metabolism, *p*-value 0.0084818; *I)* porphyrin and chlorophyll metabolism, *p*-value 0.025717; and *K)* glyoxylate and dicarboxylate metabolism, *p*-value 0.029754. **(F)** The individual metabolites in the pathway were also increased, following irradiation. Alanine, aspartate, and glutamate metabolism was also affected. Similar to the sham irradiation *versus* 8-Gy irradiation, the arginine biosynthesis and glutamine and glutamate metabolism pathway were also affected.

Analysis of the effects of radiation in the plasma in the presence of HU revealed several pathways. Within the HU groups, comparing sham irradiation *versus* 3-Gy irradiation, the phenylalanine, tyrosine, and tryptophan biosynthesis pathways were affected (Figure 7A). The individual metabolites in the pathway are shown in Figure 7B, which were increased following irradiation. The phenylalanine, tyrosine, and tryptophan biosynthesis pathways were also affected comparing sham irradiation *versus* 8-Gy irradiation (Figure 7C). The individual metabolites in the pathway were also increased, following irradiation (Figure 7D). The arginine biosynthesis and glutamine and glutamate metabolism pathways were also affected but with a much lower impact. The phenylalanine, tyrosine, and tryptophan biosynthesis pathways were also most clearly affected comparing sham irradiation *versus* 10-Gy irradiation (Figure 7E). The individual metabolites in the pathway were also increased, following irradiation (Figure 7F). The alanine, aspartate, and glutamate metabolism pathway was also affected. Similar to sham irradiation *versus* 8-Gy irradiation, the arginine biosynthesis and glutamine and glutamate metabolism pathways were also affected.

**FIGURE 8:**
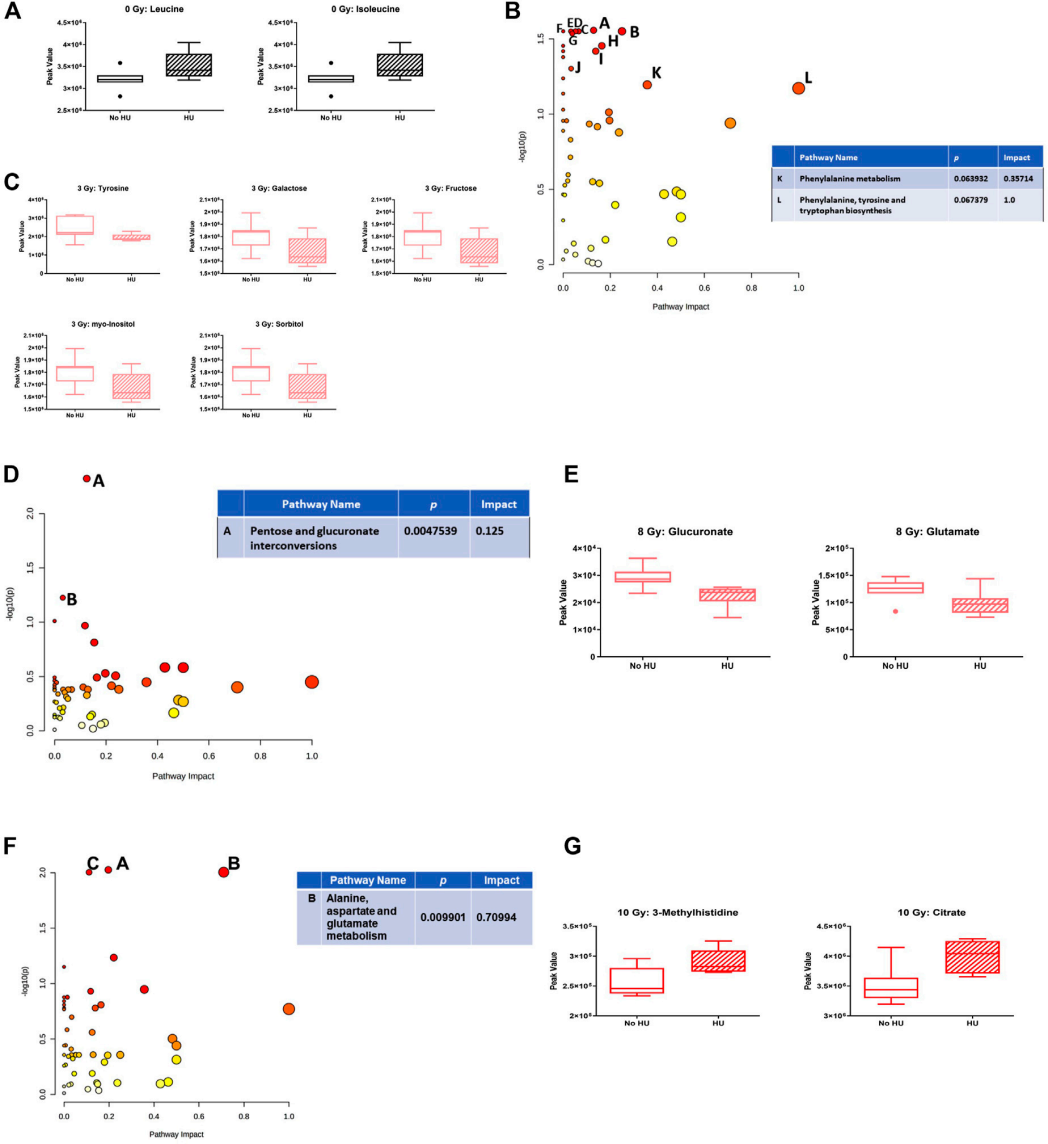
Effects of HU on metabolic pathways in plasma at each radiation dose. **(A)** In sham-irradiated animals, plasma levels of leucine and isoleucine were increased under the HU condition. **(B)** In animals irradiated with 3 Gy, the phenylalanine, tyrosine, and tryptophan biosynthesis pathways were affected by HU. **(C)** In addition to glucose-6-phosphate and glucosamine, the metabolites were decreased under the HU condition. **(D)** In animals irradiated with 8 Gy, the pentose and glucuronate interconversions pathway was affected. *B*: Butanoate metabolism pathway. **(E)** Plasma levels of glucuronate and glutamate were reduced. **(F)** In animals irradiated with 10 Gy, the alanine, aspartate, and glutamate metabolism pathway was affected. The citrate cycle (TCA cycle) (*A*; *p*-value is 0.009424) and glyoxylate and dicarboxylate metabolism (*C*: *p*-value 0.0099177) pathways were also affected but with a lower impact. **(G)** Plasma levels of 3-methylhistidine and citrate under the HU condition were increased.

We next assessed the effects of HU within each radiation dose. In sham-irradiated animals, no pathway was affected, but plasma levels of leucine and isoleucine were increased under the HU condition (Figure 8A). In animals irradiated with 3 Gy, the phenylalanine, tyrosine, and tryptophan biosynthesis pathways were affected by HU (Figure 8B). In addition to glucose-6-phosphate and glucosamine, the metabolites were decreased under the HU condition (Figure 8C). In animals irradiated with 8 Gy, the pentose and glucuronate interconversion pathway was affected to a certain extent (Figure 8D), with reduced plasma levels of glucuronate and glutamate (Figure 8E). In animals irradiated with 10 Gy, the alanine, aspartate, and glutamate metabolism pathway was affected (Figure 8F), and increased plasma levels of 3-methylhistidine and citrate were observed under the HU condition (Figure 8G).

### 3.5 Regression analysis of individual metabolites and select behavioral or cognitive measures

We used univariate linear regression analyses stratified by radiation exposure and HU condition, with Z-scores for behavioral measures as the dependent variable and the metabolite value as the independent variable, to identify potential plasma biomarkers of radiation exposure or the HU condition on behavioral or cognitive performance. We selected those metabolites that were most consistently included in the models. Table 1 lists all behavioral measures used for the regression analysis in the current study, and the footnote describes the comparison of this list with that of our earlier study.

Table 2 shows 27 plasma metabolites that were most consistently included in the models (at least four times) and which of the 13 behavioral measures they were related to. Most associations between metabolites and behavioral measures were positive, as indicated in green in Table 2. Two metabolites were negatively associated with behavioral measures (indole-3-acetate and trimethyllysine), as indicated in red in Table 2. Two metabolites, indoxyl sulfate and lauric acid, were both positively and negatively associated with behavioral measures, as indicated in blue in Table 2. For indoxyl sulfate, only one negative association was observed (distance moved on day 2 of open-field testing), while for lauric acid, only one positive association was observed (ratio times in the open arms in the elevated plus maze).

**TABLE 2:** Association of specific metabolites with behavioral measures^a^.

Non-HU/HU	Dose (Gy)	Metabolite^b^	N^c^	Significant positive associations	Significant negative associations
Non-HU	0	Asparagine	4	no2_cent_time, no2_tot_dur_objs, of2_percent_cent, and of2_tot_dist	
Non-HU	0	4_Hydroxy_d_proline	4	no1_cent_time, no1_tot_dist, no1_tot_dur_objs, and of2_tot_dist	
Non-HU	0	Galactarate	6	no1_cent_time, no1_tot_dist, no1_tot_dur_objs, no2_tot_dist, of2_percent_cent, and of2_tot_dist	
Non-HU	0	Phosphorylcholine	4	no1_tot_dist, no1_tot_dur_objs, of2_percent_cent, and of2_tot_dist	
Non-HU	0	Proline	6	no2_cent_time, no2_tot_dist, no2_tot_dur_objs, of1_percent_cent, of2_percent_cent, and of2_tot_dist	
Non-HU	0	Saccharic_acid (glucaric acid)	6	no1_cent_time, no1_tot_dist, no1_tot_dur_objs, no2_tot_dist, of2_percent_cent, and of2_tot_dist	
Non-HU	0	Serine	4	no1_tot_dist, no1_tot_dur_objs, of2_percent_cent, and of2_tot_dist	
Non-HU	0	Succinate	7	epm_ratio_open_time, no2_cent_time, no2_tot_dist, no2_tot_dur_objs, of1_percent_cent, of2_percent_cent, and of2_tot_dist	
Non-HU	0	3_Phospho_glyceric_acid	4	no2_cent_time, no2_tot_dist, no2_tot_dur_objs, and of1_percent_cent	
Non-HU	0	4_Hydroxy_l_proline	5	epm_ratio_open_time, no2_cent_time, no2_tot_dist, no2_tot_dur_objs, and of2_tot_dist	
Non-HU	0	5_Aminolevulinic_acid	4	no1_cent_time, no1_tot_dist, no1_tot_dur_objs, and of2_tot_dist	
Non-HU	0	5_Hydroxylysine	4	no2_cent_time, no2_tot_dist, no2_tot_dur_objs, and of1_tot_dist	
Non-HU	3	Homoserine	4	anh_percent_change_d1_ave, no1_tot_dist, no2_tot_dist, and of1_percent_cent	
Non-HU	3	Methionine	4	anh_percent_change_d1_ave, no1_tot_dist, no2_tot_dist, and of1_percent_cent	
Non-HU	3	1_Aminocyclopropanecarboxylic_acid	4	no1_cent_time, no1_tot_dist, no1_tot_dur_objs, and of2_percent_cent	
Non-HU	8	Indoxyl_sulfate	4	anh_percent_change_d1_ave, no2_cent_time, no2_di, and no2_tot_dur_objs	
Non-HU	8	Lauric_acid	4	epm_ratio_open_time	anh_percent_change_d1_ave, no1_tot_dist, and no2_tot_dist
Non-HU	8	Malate	5	anh_percent_change_d1_ave, no2_cent_time, no2_di, no2_tot_dur_objs, and of2_tot_dist	
	8	5_Methylcytosine	5	anh_percent_change_d1_ave, no2_cent_time, no2_di, no2_tot_dist, and no2_tot_dur_objs	
HU	0	Indole_3_acetate	4		no1_cent_time, no1_tot_dist, no1_tot_dur_objs, and of2_tot_dist
HU	3	Erucic_acid	5	anh_percent_change_d1_ave, no1_cent_time, no1_tot_dist, no1_tot_dur_objs, and of2_tot_dist	
HU	3	Gluconic_acid	6	no1_cent_time, no1_tot_dist, no1_tot_dur_objs, no2_cent_time, no2_tot_dur_objs, and of1_tot_dist	
HU	3	Heptadecanoate	7	no1_cent_time, no1_tot_dist, no1_tot_dur_objs, no2_cent_time, no2_tot_dist, no2_tot_dur_objs, and of1_tot_dist	
HU	3	LysoPA (18:1) (0/0:0	5	no1_cent_time, no1_tot_dist, no2_cent_time, no2_tot_dist, and no2_tot_dur_objs	
HU	3	Palmitate	6	no1_cent_time, no1_tot_dist, no1_tot_dur_objs, no2_cent_time, no2_tot_dist, and no2_tot_dur_objs	
HU	3	Trimethyl lysine	6		no1_cent_time, no1_tot_dist, no1_tot_dur_objs, no2_cent_time, no2_tot_dur_objs, and of1_tot_dist
HU	8	Indoxyl_sulfate	4	no1_cent_time, no1_tot_dist, and no1_tot_dur_objs	of2_tot_dist

^a^ *of*, open field; *no*, open field with objects; *epm*, elevated plus maze; *anh*, anhedonia test; *tot dist*, total distance moved; *cent time*, time spent in the center of the enclosure; *tot dur obj*, total time spent exploring the objects; *anh percent change d1 ave*, the percentage of M&Ms consumed on days 2 and 3 compared to day 1.

^b^ Metabolites positively associated with behavioral measures are indicated in green; metabolites negatively associated with behavioral measures are indicated in red; and metabolites positively and negatively associated with behavioral measures are indicated in blue.

^c^ N of significant associations.

In animals that received HU, eight metabolites were related to behavioral and cognitive measures. In HU animals that were sham-irradiated, only one metabolite was related to behavioral and cognitive measures, i.e., indole-3-acetate. In HU animals irradiated with 3 Gy, six metabolites were related to behavioral and cognitive measures, namely, erucic acid, gluconic acid, heptadecanoate, lysoPA [18–1 (9z)-0–0], palmitate, and trimethyl lysine.

In the absence of HU, 18 metabolites were related to behavioral and cognitive measures. Remarkably, no overlap in metabolites related to behavioral and cognitive measures was observed in animals that received HU compared with those that did not receive HU. In sham-irradiated animals which did not receive HU, 11 metabolites were related to behavioral and cognitive measures: asparagine, cis-4-hydroxy-d-proline, galactarate, phosphorylcholine, proline, saccharic acid (glucaric acid), serine, succinate, x3-phopshoglyceric acid, 4-hydroxyl-l-proline, and 5-aminolevulinic acid. All metabolites that were related to behavioral measures were only observed under one HU condition and in animals that received one radiation dose.

In the absence of HU, in animals that were irradiated with 3 Gy, three metabolites were related to behavioral and cognitive measures: homoserine, methionine, and 1-aminocyclopropanecarboxylic acid.

In the absence of HU, in animals irradiated with 8 Gy, five metabolites were related to behavioral and cognitive measures: indoxyl sulfate, lauric acid, malate, malate, and 5-methylcytosine.

## 4 Discussion

In this study, we characterized the behavioral and cognitive performance of male Fischer rats after sham irradiation or total body irradiation with photons, in the absence and presence of HU. In general, the effects of HU were more pronounced than those of photon irradiation. In the open-field test, HU-treated animals moved less and froze more than non-HU animals. In the open field containing objects, animals irradiated with 8 Gy moved less than the sham-irradiated animals, and only sham-irradiated rats moved more on day 2 than on day 1. In contrast, under the HU condition, animals irradiated with 3 or 8 Gy moved more on day 2 than on day 1. The analysis of anhedonia in the M&M test showed that non-HU sham-irradiated rats and rats irradiated with 8 Gy consumed more M&Ms on day 3 than on day 1. The analysis of the percentage of consumption on days 2 and 3 compared to day 1 showed an effect of radiation with a lower percentage of M&M consumption in irradiated rats than in sham-irradiated rats. In addition, HU rats irradiated with 3 Gy showed a higher percentage of M&M consumption on day 3 than on day 2. An effect of HU on the measures of anxiety in the elevated plus maze was observed, with the ratio of time spent in the open arms and time spent in the center of the elevated plus maze being lower in HU animals than in non-HU animals. Analysis of the effects of radiation in the plasma in the absence of HU showed that the phenylalanine, tyrosine, and tryptophan biosynthesis pathway was affected comparing sham irradiation *versus* 10-Gy irradiation; most plasma metabolite (except phosphatidate) levels were increased by 10-Gy radiation. The analysis of the effects of radiation in the plasma in the presence of HU showed that, comparing sham irradiation *versus* 3-, 8-, or 10-Gy irradiation, the phenylalanine, tyrosine, and tryptophan biosynthesis pathways were mostly affected, with an increase in metabolites in this pathway. The arginine biosynthesis and glutamine and glutamate metabolism pathways were also affected but with a much lower impact. The analysis of the effects of HU within each radiation dose showed that in animals irradiated with 3 Gy, the phenylalanine, tyrosine, and tryptophan biosynthesis pathways were affected; metabolite levels were lower under the HU condition (except for glucose-6-phosphate and glucosamine). In animals irradiated with 8 Gy, the pentose and glucuronate interconversion pathway was affected by HU to a certain extent; plasma levels of glucuronate and glutamate were reduced. In animals irradiated with 10 Gy, the alanine, aspartate, and glutamate metabolism pathway was affected; plasma levels of 3-methylhistidine and citrate were increased under the HU condition.

These data show that pathways for phenylalanine, tyrosine, and tryptophan metabolism and phenylalanine biosynthesis are substantially changed, following photon irradiation and HU in animals irradiated with 3 Gy. Remarkably, the phenylalanine, tyrosine, and tryptophan metabolism pathway was also mostly affected by simulated space radiation and by HU in the plasma, hippocampus, and cortex of WAG/Rij rats (Raber et al., 2021). Tryptophan is a precursor for the indolamine neurotransmitter serotonin, and tyrosine is a precursor for the catecholamine neurotransmitters dopamine, norepinephrine, and epinephrine. Tryptophan and tyrosine play a role in executive function (Aquili, 2020), which is negatively affected by X-ray irradiation (Hienz et al., 2008; Davis et al., 2014; Tang et al., 2022).

In HU animals, plasma tryptophan levels were increased, following 3- and 8-Gy irradiation. A functional deficit of tryptophan and disorders involving the hypothalamic–pituitary–adrenal axis are linked to the pathophysiology of severe depression (Begolly et al., 2017). Consistent with this notion, dexamethasone suppressed the availability of tryptophan, and major depressed patients with melancholia have lower tryptophan levels than those with minor depression (Begolly et al., 2017). In addition, tryptophan is the precursor of serotonin, and increased dietary tryptophan levels suppressed post-stress plasma glucocorticoid levels (Hoglund et al., 2019). Therefore, it is possible that increased plasma tryptophan levels in HU animals following 3- and 8-Gy irradiation might be a compensatory response and contribute to the increased activity in these two groups in activity levels on day 2 in the open field compared to that on day 1, as observed in non-HU sham-irradiated animals. As HU was started 5 days prior to the radiation exposure, the HU challenge might have served as a preconditioning challenge, mitigating the effects of these two radiation doses. As this was not observed in HU animals irradiated with 10 Gy, this dose might be too high to be compensated for by HU.

In the current study, plasma was analyzed 9 months following sham irradiation or irradiation in the absence or presence of HU and 7 months after the behavioral and cognitive testing. Together with our earlier study in WAG/Rij rats, following simulated space radiation in the absence and presence of HU, the metabolic pathways affected by HU, and, to a lesser extent, by photon irradiation, and the relationships between the behavioral and cognitive measures and individual metabolite levels in plasma, support that it is feasible to develop stable long-term biomarkers of the response to HU and of behavioral and cognitive performance.

HU had detrimental effects on the activity and freezing levels in the open field and the measures of anxiety in the elevated plus maze. These data are consistent with detrimental effects of simulated microgravity on 3-dimensional visuospatial tuning and orientation of mice (Oman, 2007). In the current study, HU sham-irradiated Fischer rats showed spatial habituation learning in the open field and moved less on day 2 than on day 1 in the open field. In contrast, HU sham-irradiated WAG/Rij rats showed impaired spatial habituation learning (Raber et al., 2021). These data suggest that Fischer rats might be less susceptible to HU than WAG/Rij rats. However, we recognize that as Fischer rats received HU at Wake Forest University while WAG/Rij rats received HU at the Brookhaven National Laboratory, we cannot exclude that environmental differences in the housing of the animals at the two institutions or differences in shipping the animals to and from these institutions might have contributed to these divergent findings.

In general, the measures of anxiety of the rats in the elevated plus maze were high (ratio times between 0.01 and 0.08). Similarly, the rats spent little time in the center of the open field during the second open-field test (between 10 and 52 s). These relatively high measures of anxiety might have contributed to only 38% of the rats exploring the objects. As the objects used in this study are routinely used successfully for testing object recognition in mice, it seems unlikely that the objects used were anxiety-provoking and more likely that the rats had, in general, elevated anxiety levels.

Indole acetic acid is an intestinal bacterium-derived tryptophan metabolite. Injecting indole into the cecum of rats decreased activity levels, and increasing indole in germ-free rats by colonizing them with the indole-producing *Escherichia coli* caused enhanced anxiety levels in the open field and elevated plus maze (Jaglin et al., 2018). Consistent with these data, in sham-irradiated rats exposed to the HU condition, indole-3-acetate levels were negatively associated with activity measures (activity levels during the second open-field testing and the first test in the open field containing objects) and reduced anxiety measures (time spent in the center of the first open-field test containing objects and time spent exploring objects in that test) in the current study. However, in a mouse model of unpredictable chronic mild stress (UCMS), administration of indole acetic acid (50 mg/kg for 5 weeks) reduced anxiety-like behavior, increased the expression of the brain-derived neurotrophic factor, and reversed the UCMS-induced imbalance of microbial indole metabolites in the colon (Chen et al., 2022). These data suggest that while HU is an environmental stressor, it was a transient stressor, and therefore, the associations of these metabolites with behavioral measures might have been negative.

In humans, trimethyl lysine was identified as a strong predictor of the risk of developing cardiovascular disease (Li et al., 2018). Trimethyl lysine is a precursor of carnitine; the buildup of this metabolite could indicate reduced beta-oxidation and oxidative phosphorylation and could lead to fatigue/reduced activity. Consistent with this, the levels of trimethyl lysine in HU animals irradiated with 3 Gy were negatively associated with the measures of activity (first open-field test without objects and first open-field test with objects) and reduced anxiety (time spent in the center in the first and second open-field tests containing objects, and time spent exploring objects in these two tests) in this study.

The mono-unsaturated omega-9 fatty acid erucic acid (3 mg/kg) enhanced cognitive performance in drug-naïve mice and ameliorated scopolamine-induced memory impairments associated with increased phosphorylation of poly-phosphatidylinositide 3-kinase (PI3K), protein kinase C-zeta, extracellular signal-regulated kinase, cAMP-response element-binding protein, and additional protein kinase B in the hippocampus (Kim and Al, 2016). Erucic acid showed protective effects in the brain and the intestines of a rotenone-treated zebrafish model of Parkinson’s disease and improved activity levels (Ünal and Al, 2022). Other beneficial effects of erucic acid include antibacterial and antiviral activities, as well as cytotoxic anticancer activity (Galanthy et al., 2023). In addition, levels of erucic acid in HU animals irradiated with 3 Gy were positively associated with the change in anhedonia compared to day 1, the activity levels during the second open-field test without objects and the time spent in the center, and activity levels and time spent exploring objects in the first open-field test containing objects. However, animal studies indicate that erucic acid might cause cardiotoxicity as well [for a review, see (Galanthy et al., 2023)].

Gluconic acid, which, once fermented to butyrate in the gut, can improve gut function, increased feed intake and improved feed utilization in weaned piglets (Michiels et al., 2023). Considering the gut–brain axis (Willyard, 2021), it is remarkable that gluconic acid levels in HU animals irradiated with 3 Gy were positively associated with activity (activity levels in the first open-field test without objects and first open-field test with objects) and reduced anxiety-like behavior (time spent in the center and time spent exploring objects in the first and second open-field tests containing objects) measures in the current study.

LysoPA 18:1 reduced activity levels in the open field, increased anxiety levels in the elevated plus maze, and increased depression-like behavior in the forced swim test (Castilla-Ortega et al., 2014). In contrast to this result, lysoPA 18:1 levels in HU animals irradiated with 3 Gy were positively associated with activity (distance moved in the first and second open-field test containing objects) and reduced anxiety measures (time spent in the center of the first and second open-field tests containing objects and time spent exploring the objects in the second open-field test containing objects) in the current study.

In the current study, palmitate levels in HU animals irradiated with 3 Gy were positively associated with activity (activity levels in the first and second open-field tests containing objects) and anxiety-like behavior (time spent in the center of the open field and exploring objects in the first and second open-field tests containing objects) measures. Consistent with this result, palmitate improved cognitive performance in the T-maze and sensorimotor function in the rotarod test (Moazedi et al., 2007). However, palmitate reduced the activity levels and increased anxiety levels in the elevated zero maze and reduced the time spent exploring a novel object 24 h after treatment (Moon et al., 2014). In addition, in the 6-hydroxydopamine model of PD, palmitate levels in plasma were increased and correlated with motor dysfunction (Shah et al., 2019). Furthermore, increased serum levels of palmitate promote insulin resistance and inhibited the clock function in hepatocytes by inhibiting nicotinamide adenine dinucleotide (NAD)-dependent deacetylase sirtuin 1 (SIRT1) (Tong et al., 2015).

In sham-irradiated animals without HU, plasma proline levels were positively associated with activity (activity levels during the second open-field test without and with objects) and reduced anxiety measures (time spent in the center of the first and second open-field tests without objects and second open-field test with objects, and time spent exploring the objects in the second open-field test with objects). However, plasma proline levels in humans and preclinical models were linked to depression, and proline supplementation in mice exacerbated depression (Mayneris-Perxachs et al., 2022).

L-serine reduced the measures of anxiety in the open field in wild-type and growth hormone-releasing hormone-knockout mice and mitigated cognitive impairments in the novel object-recognition test and sociability in growth hormone-releasing hormone-knockout mice (Zhang et al., 2023). L-serine was also associated with improved EEGs and reduced seizure in patients with GRIND (mutations in the N-methyl-D-aspartate (NMDA) receptor)-related disorders (Krey et al., 2022). In PD patients, D-serine adjuvant treatment reduced behavioral and motor symptoms (Gelfin et al., 2012). Consistent with these beneficial effects, proline levels in sham-irradiated animals without HU were positively associated with the activity levels and time spent in the center of the second open-field test without objects and activity levels and time spent exploring object in the first open-field test containing objects.

Chronic succinate feeding enhanced the endurance exercise of mice (Xu et al., 2022). Consistent with this result, succinate levels in sham-irradiated animals without HU were positively associated with activity (activity levels in the second open-field test without and with objects) and reduced anxiety-like behavior (ratio times in the open arms in the elevated plus maze, time spent in the center of the first and second open-field tests without objects and second open-field test with objects, and time spent exploring objects in the second open-field test with objects) levels.

5-Aminolevulinic acid inhibited oxidative stress and reduced autistic-like behavioral in prenatal valproic acid-exposed rats (Matsuo et al., 2020). Consistent with this result, levels of 5-aminolevulinic acid were positively associated with activity (activity levels during the second open-field test without objects and first open-field test with objects) and anxiety (time spent in the center and time spent exploring objects in the first open-field test with objects) levels.

1-Aminocyclopropanecarboxylic acid inhibited memory fading in naïve rats and prevented amnesia induced by ketamine or phencyclidine (Popik et al., 2014). 1-Aminocyclopropanecarboxylic acid also reduced the measures of anxiety in the elevated plus maze (Trullas et al., 1989). Consistent with these results, levels of l-aminocyclopropanecarboxylic acid in animals without HU irradiated with 3 Gy were positively associated with activity levels (activity levels during the first open-field test containing objects) and reduced anxiety (time spent in the center of the second open-field test without objects and first open-field test with objects, and time spent exploring objects during the first open-field test with objects) levels. We recognize that 1-aminocyclopropanecarboxylic acid is a plant or microbial metabolite and might be related to the rodent diet.

The intraperitoneal administration of indoxyl sulfate in mice with unilateral nephrectomy resulted in increased depression-like behavior in the forced swim and tail suspension tests, increased measures of anxiety in the open field, and cognitive impairments in the water maze (Sun et al., 2021). Chronic administration of indoxyl sulfate for 24 days in drinking water (200 mg/kg) reduced activity levels in the open field, elevated plus maze, and chimney and splash tests, and reduced spatial memory in the *t*-test (Karbowska et al., 2020). Consistent with these animal studies, indoxyl sulfate levels were increased in patients with early-stage chronic kidney disease and associated with poor executive function (Yeh et al., 2016). In contrast to these results, in animals without HU irradiated with 8 Gy, indoxyl sulfate levels were positively associated with the change in anhedonia compared to day 1, the time spent in the second open-field test containing objects, the time spent exploring objects in that test, and the discrimination index, a measure of cognitive performance.

Lauric acid improved behavioral performance, motor function, food intake, and weight in the haloperidol-induced PD rat model (Zaidi et al., 2020). In the current study, lauric acid levels were positively associated with reduced anxiety measures in the elevated plus maze (ratio times spent in the open arms) but negatively associated with the anhedonia change compared to day 1 and the activity levels in the first and second open-field tests containing objects.

This study had several limitations: 1) brain tissues could not be collected and analyzed because of COVID-19-related modified operations and travel restrictions; 2) plasma was not analyzed at earlier time points closer to the behavioral and cognitive testing; 3) the forced swim test could not be used to assess depression-like behavior in rats, making it harder to compare depression-like behavioral in this study and our previous study; and 4) no female rats were included in this study.

In summary, the detrimental effects of HU on the behavioral and cognitive performance and metabolic pathways illustrate the importance of developing mitigators to reduce the effects of microgravity on the brain function of astronauts during and following space missions. The metabolomics data, including the associations of specific metabolites in plasma, collected 7 months after behavioral testing, with behavioral measures, suggest that it will be possible to develop stable plasma biomarkers of HU and behavioral and cognitive performance that could be used for developing and testing such mitigators. There were five metabolites positively related to behavioral and cognitive measures in HU animals irradiated with 3 Gy: erucic acid, gluconic acid, heptadecanoate, lysoPA [18–1 (9z)-0–0], and palmitate. Trimethyl lysine was the only metabolite negatively related to behavioral and cognitive performance in the HU animals irradiated with 3 Gy. These data suggest that these six metabolites may be of benefit to mitigate HU- and radiation-induced behavioral alterations and cognitive injury.

## Data Availability

The original contributions presented in the study are included in the article/Supplementary Material; further inquiries can be directed to the corresponding author.
