# Supplementary material for: Effects of photon irradiation in the presence and absence of hindlimb unloading on the behavioral performance and metabolic pathways in the plasma of Fischer rats
Source: Front Physiol. 2024 Jan 8;14:1316186. doi: 10.3389/fphys.2023.1316186 (PMC10800373; doi:10.3389/fphys.2023.1316186)
Supplement: Supplementary file 1 [file Table1.DOCX]

**Supplementary figure legends**

**Suppl. Fig. 1.** Time of non-HU and HU sham-irradiated and irradiated rats spent in the center of the open field.

**Suppl. Fig. 2.** Ratio entries of non-HU and HU sham-irradiated and irradiated rats spent in the elevated plus maze.
