# Supplementary figures and images for: Effects of photon irradiation in the presence and absence of hindlimb unloading on the behavioral performance and metabolic pathways in the plasma of Fischer rats
Source: Front Physiol. 2024 Jan 8;14:1316186. doi: 10.3389/fphys.2023.1316186 (PMC10800373; doi:10.3389/fphys.2023.1316186)

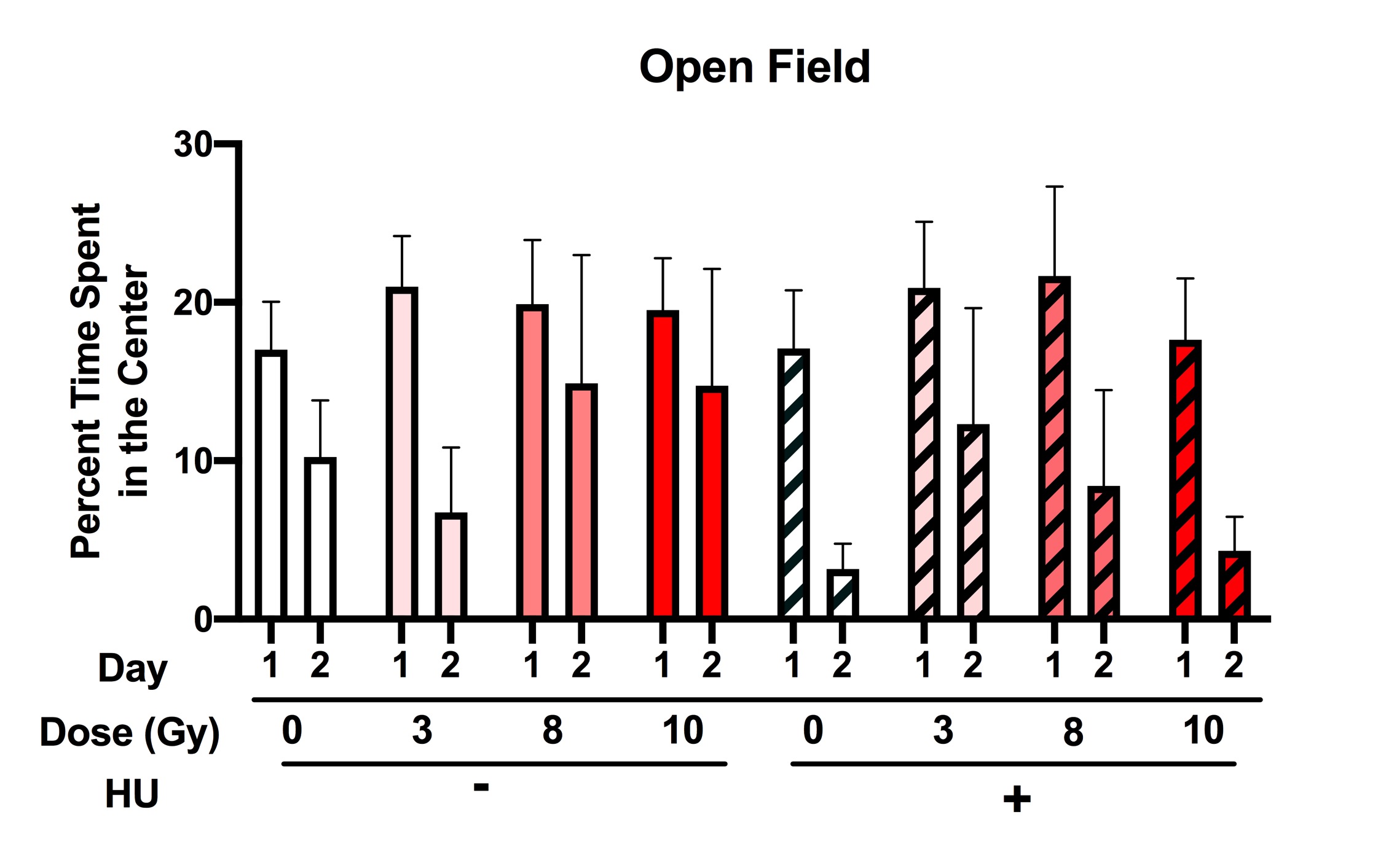

Supplement: Supplementary file 2 [file Image1.JPEG]

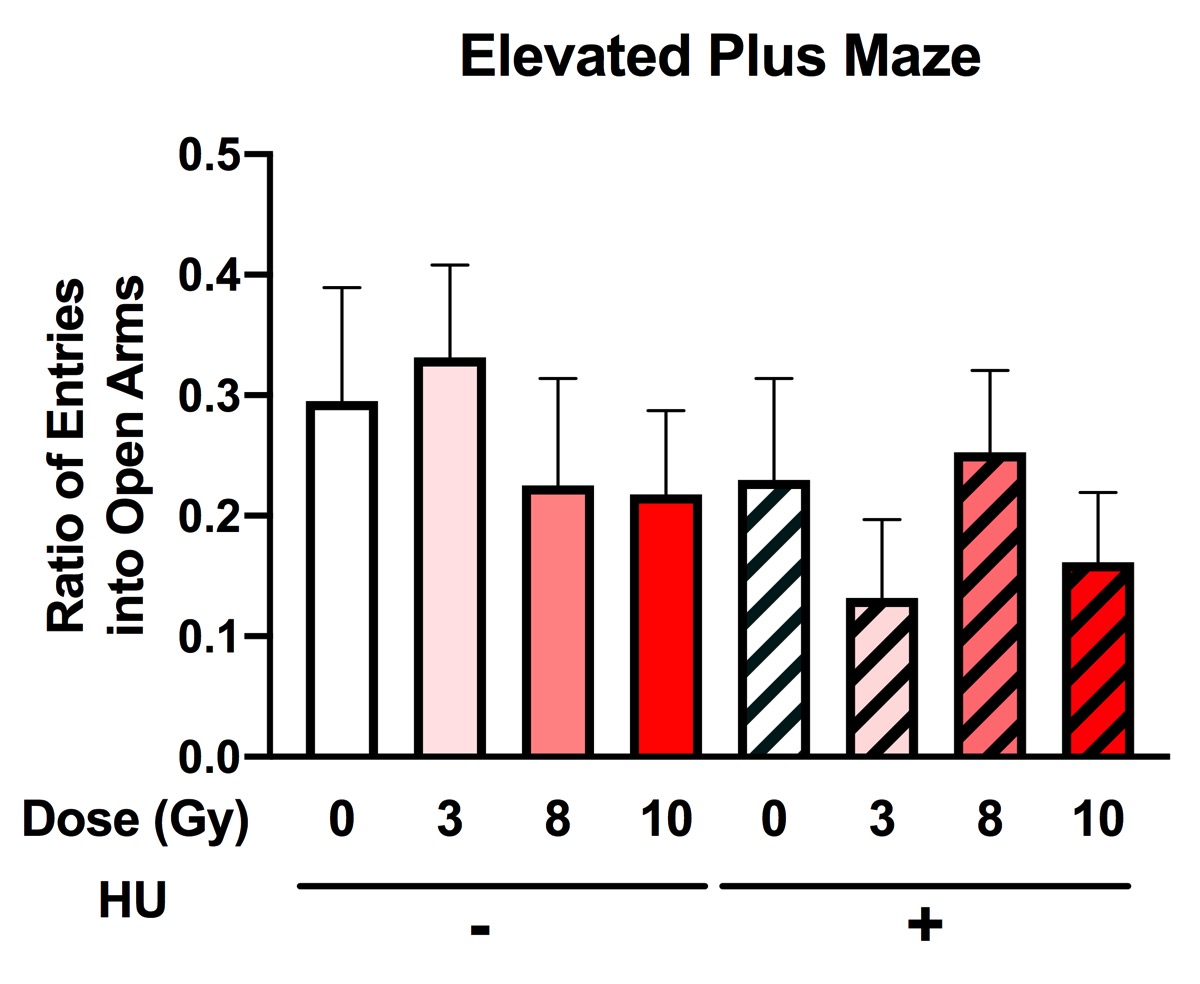

Supplement: Supplementary file 3 [file Image2.JPEG]
